# Multiscale Analysis of Solar Loading Thermographic Signals for Wall Structure Inspection †

**DOI:** 10.3390/s21082806

**Published:** 2021-04-16

**Authors:** Katherine Tu, Clemente Ibarra-Castanedo, Stefano Sfarra, Yuan Yao, Xavier P. V. Maldague

**Affiliations:** 1 Department of Chemical Engineering, National Tsing Hua University, Hsinchu 30013, Taiwan; s105032074@m105.nthu.edu.tw; 2 Department of Electrical and Computer Engineering, Laval University, Québec City, QC G1V 0A6, Canada; clemente.ibarra-castanedo@gel.ulaval.ca (C.I.-C.); Xavier.Maldague@gel.ulaval.ca (X.P.V.M.); 3 Department of Industrial and Information Engineering and Economics, University of L’Aquila, I-67100 L’Aquila, AQ, Italy

**Keywords:** infrared thermography, solar loading thermography, multiscale analysis, multidimensional ensemble empirical mode decomposition, principal component analysis

## Abstract

Infrared thermography has been widely adopted in many applications for material structure inspection, where data analysis methods are often implemented to elaborate raw thermal data and to characterize material structural properties. Herein, a multiscale thermographic data analysis framework is proposed and applied to building structure inspection. In detail, thermograms are first collected by conducting solar loading thermography, which are then decomposed into several intrinsic mode functions under different spatial scales by multidimensional ensemble empirical mode decomposition. At each scale, principal component analysis (PCA) is implemented for feature extraction. By visualizing the loading vectors of PCA, the important building structures are highlighted. Compared with principal component thermography that applies PCA directly to raw thermal data, the proposed multiscale analysis method is able to zoom in on different types of structural features.

## 1. Introduction

Active infrared thermography (AIRT) [1] has been widely applied in non-destructive testing (NDT) of structures because of its non-contact nature, easy operation, and wide inspection area. When applying AIRT, the inspected object is heated by one or multiple external energy sources and the surface temperature distribution of the target object is captured by an infrared camera in the form of a series of thermograms. The thermogram sequence recorded through time reflects both the spatial and temporal changing patterns of the surface temperature, which indicates the variations of material thermal conductivity. As a result, the surface and internal structures associated with thermal heterogeneity can be identified from the thermograms generated by AIRT.

In recent years, solar loading thermography [2] has been successfully implemented to inspect civil engineering structures, where solar irradiation and environmental temperature changes are used as the external energy source and the surface temperature evolution of the target object is recorded by an infrared camera to generate a long-term sequence of thermograms.

Emissivity is the most important parameter for obtaining the accurate temperature and it is approximately constant at a viewing angle less than 45°. In case of changing the object to detector distance, this will not affect to the temperature measurement if measurement field of view (MFOV) at the object level is smaller than the targeted object [3].

However, the impact of the emissivity variation is dramatically reduced if post-processing algorithms such as Fourier transform are applied to raw images [4]. If possible (i.e., if the conditions surrounding the facade of the building to be analyzed allow a proper measurement), the angle of 45° between the perpendicular to the facade and the thermal camera should be respected above all to understand the shape of the *thermal imprint* projected on the surface by materials having different thermal properties respect to the upper layer facing the sun [5]. Contextually, the solar height, i.e., the vertical angle formed between the collimated direction of the sun and the horizontal plane, does not have a particular impact on the measurements since the thermal images are collected over time for days [2]. Bearing this in mind, the surface temperature variations follow a sinusoidal periodic stimulation, the thermal wave intensity at the surface (*z* = 0) (with *z* = depth of penetration [m]) is dictated by the material effusivity, while the wave attenuation and speed depend on the material diffusivity.

On the one hand, thermal diffusivity provides a measure of the material’s ability to conduct heat in relation to its capacity to store it. On the other hand, thermal effusivity measures the material’s ability to exchange heat with another material with which it is in contact. This is of great interest in our case, since we investigated the periodic heating, where effusivity helps to characterize thermal interactions at the interface of two media through which heat propagates (i.e., the so-called thermal inertia). Therefore, the intensity of the heat source *I*_0_ [W/m^2^] is directly proportional to *T*(*z*,*t*), and inversely proportional both to the thermal effusivity *e* [W s^1/2^/m^2^ K] and the square root of the modulation frequency *ω* [rad/s] [6]. It is possible to conclude that the *I_0_* affects the results of the thermographic inspection in case it is not enough to generate a sufficient thermal contrast between the feature of interest and the background.

Obvious structural information can be extracted by naked-eye observation (i.e., without any advanced image processing) of the surface under analysis. Such information can be implemented in a complex numerical modelling in which the computational fluid dynamics (CFD) is at the center of the investigation of the heat transfer through multilayered materials used in civil engineering [7,8]. Although this part falls outside the purposes of the present research, the use of, e.g., Comsol Multiphysics^®^ software can be of help to determine the minimal and optimal *I*_0_ able to generate the higher thermal contrast.

This laborious part can be overcome by the use of thermographic image processing that is a necessary step to highlight the interested features [9,10,11,12,13,14,15]. Among these techniques, principal component thermography (PCT) [16] is popular because of its capability in data compression, noise reduction, and feature extraction. In recent years, extensions of PCT have been developed to further improve its performance. To list some examples, candid covariance-free incremental PCT [17] improves the calculate on of the conventional PCT, sparse PCT [18,19] better separates different types of information by introducing sparsity constraints into the loadings, sparse moving window PCT [20] pays more attention to the time-wise correlations by using the moving window technique, and generative PCT [21] adopts the state-of-the-art generative adversarial network, which is a branch of deep learning, to achieve image augmentation and enhance the detection performance of PCT. Other extensions of PCT include robust PCT [22], etc. In [2], PCT was used to analyze the thermal data collected in the experiment of solar loading thermography.

A gap between research and the practice is that most civil engineering structures are inherently of multiple spatial scales and the existing thermographic data processing methods seldom consider this. Therefore, some important structural details may be missed in the analysis. In the field of defect detection in polymer composite materials and mosaics [23,24], a nonparametric signal processing method named multi-dimensional ensemble empirical mode decomposition (MEEMD) has been found to be applicable, which separates each thermogram to several intrinsic mode functions (IMFs) according to the levels of local frequency. In doing this, the material structural information can be separated from the high-frequency noise and the low-frequency non-uniform thermal backgrounds. Consequently, the structural information of the material is extracted at different spatial scales. However, MEEMD deals with each single thermogram and results in a large number of feature images. Further analysis of the decomposition results is a time-consuming and laborious task.

In this work, a multiscale thermographic data processing framework is proposed. First, MEEMD is used to decompose each thermogram collected in the solar loading thermography experiments, which leads to a series of IMFs corresponding to different spatial scales. Then, PCA is implemented to each scale, so that the material structure information can be better extracted, and the data size can be reduced. At the same time, PCA can also be used to process the original thermograms, which is equivalent to PCT [25]. In a sense, the proposed framework can be regarded as a multiscale generalization of PCT.

The remaining sections of this paper are organized as follows. The methodologies are introduced in Section 2, including a brief introduction of solar loading thermography, the fundamentals of MEEMD and PCA, and the framework of the proposed method. Then, experimental results on wall structure inspection are presented in Section 3, which illustrate the feasibility of the proposed method. Finally, conclusions are made in Section 4.

## 2. Methodologies

### 2.1. Solar Loading Thermography

Solar loading thermography [2,26,27,28,29] is a promising NDT approach for inspecting large civil engineering constructions. In such applications, it is difficult, sometimes impossible, to thermally stimulate the tested object with an artificial energy source. Therefore, solar loading thermography takes advantage of solar irradiation as a practical alternative. Different from the energy sources adopted in conventional AIRT, solar irradiation is usually not in control of the operators. Neither the sun nor the weather is controllable. However, this does not affect the collection of thermograms that reflect the surface temperature evolution of the investigated object. As in other AIRT methods, these thermograms are recorded by an infrared camera during the testing time period, from which the surface and subsurface material structures can be inferred.

Usually, the acquired thermographic data need to be processed with signal processing, statistical, or machine learning methods for noise reduction, data compression, contrast improvement, and feature extraction. These processing steps often greatly facilitate the detection of various structural characteristics and reduce the labor and time. For example, the use of pulsed phase thermography [9] and PCT was reported in the reference paper [2].

### 2.2. Thermogram Decomposition with MEEMD

As discussed in the Introduction section, multiple spatial scales are an inherent characteristic of many civil engineering structures. As a result, solar loading thermography testing often results in thermograms containing multi-spatial scale information. In such cases, MEEMD is a powerful tool to achieve signal decomposition of each thermogram.

The basis of MEEMD is empirical mode decomposition (EMD) [30]. Similar to Fourier analysis [31] and wavelet analysis [32], EMD aims to partition a series of images into several component signals according to different frequencies. Compared with other methods such as wavelets, EMD is a nonparametric adaptive data analysis method whose results do not depend on the selection of parameters. Moreover, EMD is capable of dealing with nonlinear and nonstationary signals. The conventional EMD works on one-dimensional signals, which breaks down a time series x in the following way.

(1) $$x\left(t\right)=\overset{J}{\underset{j=1}{\sum }}c_{j}\left(t\right)+r\left(t\right)$$

where $c_{j}$ is the *j-*th component signal, i.e., an IMF, separated from the original signal x, which is sorted by frequency, *J* is the total number of IMFs, and r is the monotonic residue. The IMFs should satisfy the following requirements [33].

(a) “The number of extrema and the number of zero-crossings must either be equal or differ at most by one.”
(b) “At any point, the mean value of the upper and lower envelopes defined by the local maxima and local minima is zero.”

The procedure for extracting an IMF is called sifting, which can be described as follows.

(1) Let s(t)=x(t).
(2) Identify all the local extrema in s(t).
(3) Generate the upper envelope by connecting all the local maxima with a cubic spline.
(4) Generate the lower envelope by connecting all the local minima with another cubic spline.
(5) Calculate the mean of the two envelops and denote it as m(t). The frequency of m(t) is lower than that of the original signal.
(6) Subtract m(t) from S(t) to obtain an oscillatory signal h(t).
(7) Check if h(t) satisfies the requirements (a) and (b) for an IMF.
(8) If the conditions of IMF are not satisfied, let S(t)=h(t) and repeat the above steps.
(9) Otherwise, an IMF is obtained as $c_{1}\left(t\right)=h\left(t\right)$.
(10) Update s(t) with the residue between the original signal x(t) and the sum of all obtained IMFs. Repeat the previous steps to find out all the IMFs.
(11) Terminate the iterative procedure if the residue r(t) becomes a monotonic function.

The performance of the conventional EMD is often degraded because of the mode mixing problem [32]. In detail, an IMF calculated by EMD may consist of signals of different frequencies, while the signals at the same scale may occur in different IMFs. The main reason of mode mixing is the intermittency in the signal.

Ensemble empirical mode decomposition (EEMD) [34] is a solution to the mode mixing problem, which adds white noise with a finite amplitude to the original signal before conducting EMD and repeats the procedure for multiple times. Finally, a “true” IMF can be obtained by averaging the IMFs at the corresponding scale obtained in different EMD trails. The averaging cancels both the mode mixing effect and the noise added to the signal, leading to an improved result.

**Figure 1:**
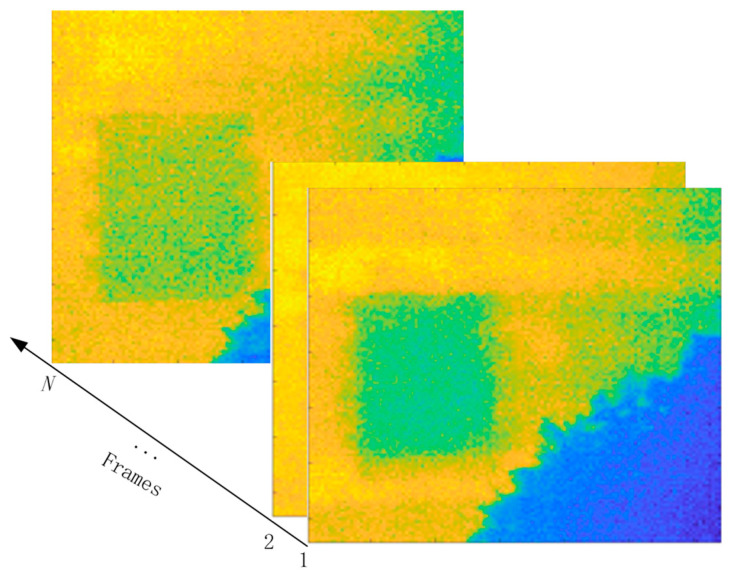
Structure of thermographic data.

MEEMD [35] is an extension of EEMD to multi-dimensional applications, which is used in this study to decompose each thermogram collected in the solar loading thermography testing. Herein, each thermogram is treated as a two-dimensional signal whose *x*- and *y*-dimensions correspond to the horizontal and vertical pixels, respectively. The number of thermograms collected in the experiment is *N*. Figure 1 illustrates the thermographic data structure. The procedure of implementing MEEMD is as follows.

First, EEMD is applied to each *x*-direction signal, i.e., each row of a thermogram, which results in $J_{x}-1$ IMFs and one residue trend. Consequently, $J_{x}$ images are acquired, each of which is composed of the *i*-th IMF or residue calculated from each row signal, $i=1,\ldots ,J_{x}$. In other words, the original thermogram is decomposed to $J_{x}-1$ IMF images and a residue image.

Then, EEMD is applied again to the *y*-direction of each IMF and residual image obtained in the previous step. Each of the $J_{x}$ images are further decomposed to $J_{y}$ sub-images. Therefore, totally $J_{x}$ times $J_{y}$ sub-images are obtained. Denote each sub-image as $H_{j,i}$, where $j=1,\ldots ,J_{x}$ and $i=1,\ldots ,J_{y}$.

In the third step, these sub-images are combined to *K* feature images, where $K=\min(J_{x},J_{y})$ and the *k*-th feature image $C_{k}$ is calculated as

(2) $$C_{k}=\overset{J_{y}}{\underset{j=k}{\sum }}H_{k,j}+\overset{J_{x}}{\underset{j=k+1}{\sum }}H_{j,k}$$

These feature images correspond to different spatial frequencies, which provide a multiscale view of the thermograms collected in the solar loading thermography experiments. Each feature image has the same resolution as the original thermogram.

More mathematics of the MEEMD method can be found in the reference [35].

### 2.3. PCA for Feature Extraction at Each Spatial Scale

By conducting MEEMD, *K* feature images are decomposed from a thermogram. Considering that there is a total of N thermograms collected in the experiments, the total number of the feature images obtained by conducting MEEMD equals to N times K. It is common that N is equal to several hundred. Therefore, the number of component images is usually very large. The manual checking of all the component images is laborious, time-consuming, and error prone. Therefore, it is necessary to use a dimensionality reduction technique, such as PCA, for data compression and feature extraction at each spatial scale.

Before performing PCA, the feature images corresponding to the *k*-th spatial scale are unfolded to a two-dimensional data matrix $X_{k}$ (k=1,…,K). In detail, the feature images are vectorized and stored in a matrix as the row vectors. Because there are *K* spatial scales according to the results of MEEMD, hence *K* unfolded data matrices are obtained, each of which has the dimensions of N×M. Herein, M is the total number of pixels in each thermogram (or feature image). Similarly, the data unfolding step can also be implemented to the original thermograms, with the unfolded matrix denoted as $X_{0}$.

Then, PCA is used to summarize the material structural information contained in each $X_{k}$ (k=0,…,K). Without loss of generality, it is assumed that the unfolded data matrix $X_{k}$ has been normalized before conducting PCA. The mathematical expression of PCA is as follows.

(3) $$X_{k}=T_{k}P_{k}^{T}$$

where $T_{k}$ is the score matrix each column of which is a vector of principal component (PC), and $P_{k}$ is the loading matrix describing the transformation relationship between $X_{k}$ and $T_{k}$.

The problem described in Equation (3) can be solved by several algorithms [36], including eigenvalue decomposition, singular value decomposition, etc. The first PC vector, i.e., the first column in score matrix $T_{k}$, captures a large amount of the variability in the dataset, i.e., $X_{k}$. The second PC locates in the orthogonal space of the first one, which explains the largest fraction of the variance not explained by the first PC, and so on and so forth. In this way, the first several PCs extract most of the systematic variation information contained by the dataset, which are most important. The corresponding loading vectors, i.e., the columns in matrix $P_{k}$, are orthonormal to each other. Physically, the loadings represent the correlation between the pixels in the images. Obviously, the first several loadings, i.e., the first several columns in $P_{k}$, contain more useful information than the remainders, which are then reshaped and visualized.

In the visualization step, a loading vector with the dimensions M×1 is reshaped to a matrix with the same dimensions as the original thermogram (or feature image). Then, this matrix is visualized as a heat map. In such a loading image, the highly correlated pixels, usually corresponding to the areas with similar physical properties, i.e., have similar colors, while the areas with different thermal behaviors often have diverse colors. Therefore, the generation of the loading images facilitate the identification of material structural properties.

Supposing that A loading images are plotted at each spatial scale, the total number of all loading images is A times K. Because A is usually much smaller than N, it is more convenient to inspect the loading images than the MEEMD feature images and the original thermograms.

**Figure 2:**
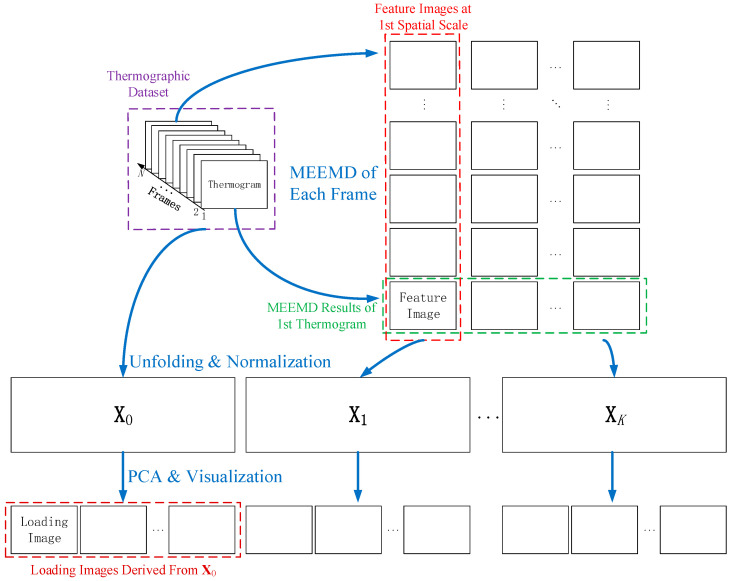
Procedure of multiscale thermographic data processing.

The entire procedure of the proposed multiscale thermographic data processing framework is shown in Figure 2.

## 3. Experimental Results

### 3.1. Solar Loading Thermography Experiment for Inspecting a Building Wall

**Figure 3:**
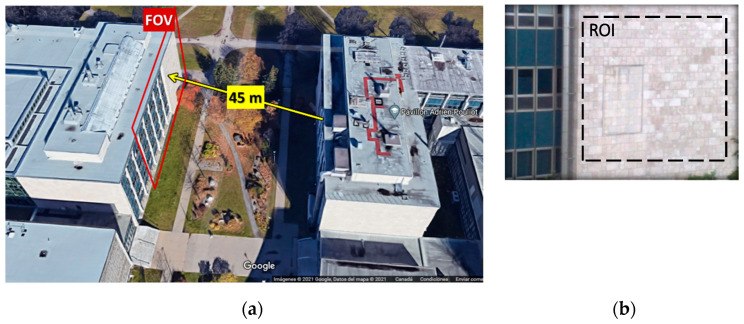

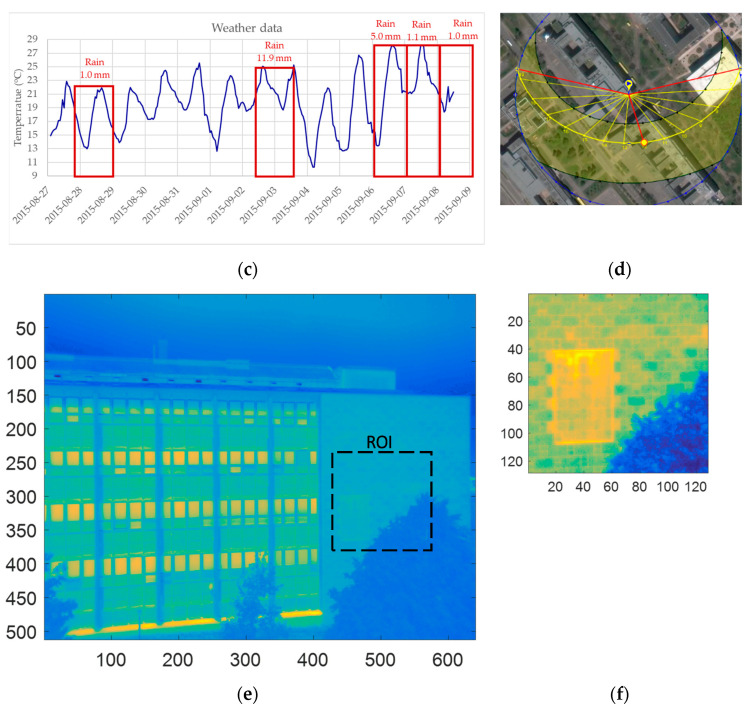
View of inspected wall: (**a**) 3D view from Google Maps highlighting the FOV and the distance between the camera (3rd floor of the right building) and the wall (left building); (**b**) photo of the ROI; (**c**) weather data during full survey period (source: Canadian Weather website: https://weather.gc.ca/canada_e.html (accessed on 3 March 2021); (**d**) satellite view of the building showing the sun path for 1 September 2015 (source http://www.sunearthtools.com (accessed on 3 March 2021)); (**e**) thermogram taken 1 September 2015 at noon showing the full FOV; and (**f**) ROI from (**e**).

The thermograms used to illustrate the proposed method were collected in a solar loading thermography experiment carried out at Laval University, Quebec City, QC, Canada in the summer 2015. In this experiment, the investigated object is a building wall, which is composed of concrete blocks with a limestone facing. Figure 3 shows a photo of the inspected wall, from which the surface structures can be observed.

A long-wave infrared microbolometer (FLIR A65, 7.5 to 13 μm, 640 × 512 pixels) was used for collecting the thermographic data using a 13 mm lens, which provided a field-of-view (FOV) of 45° × 37°. The camera was located on the 3rd floor of the building facing the wall at an approximated distance of 45 m as seen in Figure 3a. Hence, a total area of approximately 38 × 30 m^2^ was observed, although the region-of-interest (ROI) in this study was limited to a portion of the wall of approximately 128×128 pixels (see Figure 3b), i.e., 7.5 × 7.5 m^2^ or approximately 6 cm per pixel. Figure 3c shows the weather data from the Canadian Weather website for the full duration of the survey.

The wall is facing South-East as shown in Figure 3d, which presents a satellite view and the sun path corresponding to 1 September 2015 at noon (selected arbitrarily). As can be seen, for this specific day, the wall was exposed to the sun from 6:00 a.m. to 7:30 p.m. with changing angles of incidence. Solar irradiation varied slightly during previous and subsequent days.

Figure 3e presents a thermogram (corresponding to 1 September 2015 at noon) showing the full frame (640 × 512 pixels) FOV. The corresponding ROI (128 × 128 pixels) is presented in Figure 3f. The acquisition rate of the thermograms was 1/300 Hz, i.e., one thermogram was collected every five minutes.

**Figure 4:**
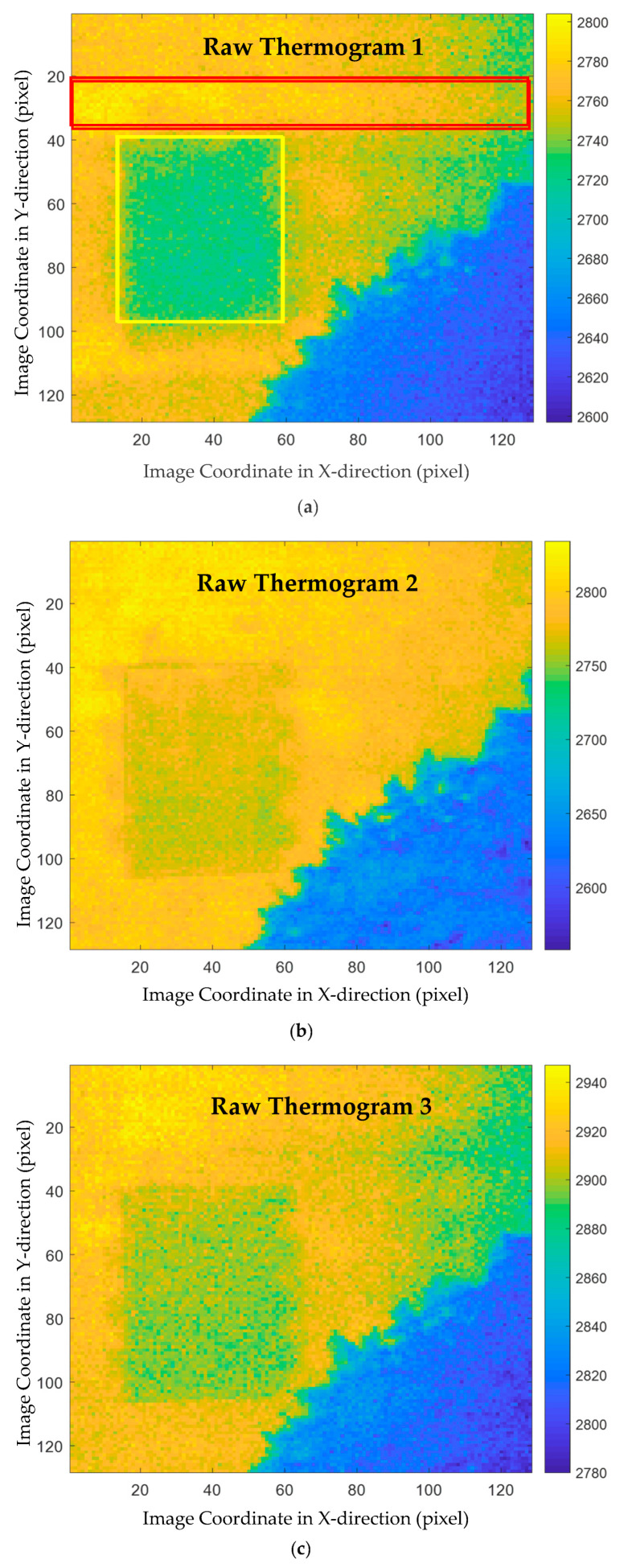
Thermograms acquired at several arbitrarily selected time points: the positions of a floor slab and a sealed door are marked in (**a**), while a tree in the foreground can be observed in all images (**a**–**c**).

In detail, the measurements were taken every five minutes during 27 August to 8 September 2015. To illustrate the proposed method, the original thermograms were down-sampled by taking one of every 100 frames. Therefore, a total of 38 thermograms were used for analysis. Figure 4 shows some arbitrarily chosen thermograms that were recorded at different time. The color bars indicate the gray levels (not the temperature since data was not calibrated). In these thermograms, the details of the surface structure of this wall, such as the tiles, are not very clear and almost invisible in these raw images. The region with lower gray levels at the right bottom of each thermogram corresponds to a tree in the foreground. Therefore, this region has a lower intensity levels than the regions not covered by the tree. In the first subplot of Figure 4, two internal structure signatures are marked, where the area in the red box corresponds to a floor slab and a sealed door is surrounded by a yellow box. Obviously, the sealed door is easy to identify because the corresponding area often has a lower surface temperature than its surroundings, while the boundary of the slab is not distinguishable in many thermograms. In addition, the right part of the ROI sometimes has a lower temperature than the left part, although there is no special inner structure therein. It is desired to apply the advanced thermographic data decomposition and analysis methods to improve the identification of the civil engineering structures that are of interest.

### 3.2. Decomposition of Thermograms

To illustrate the feasibility of the proposed multiscale thermographic data analysis method, a sequence of 38 thermograms was processed. First, each of these thermograms were decomposed by implementing MEEMD. Five feature images were obtained from the decomposition results of each thermogram, each of which contains some structure features at a different spatial scale.

**Figure 5:**
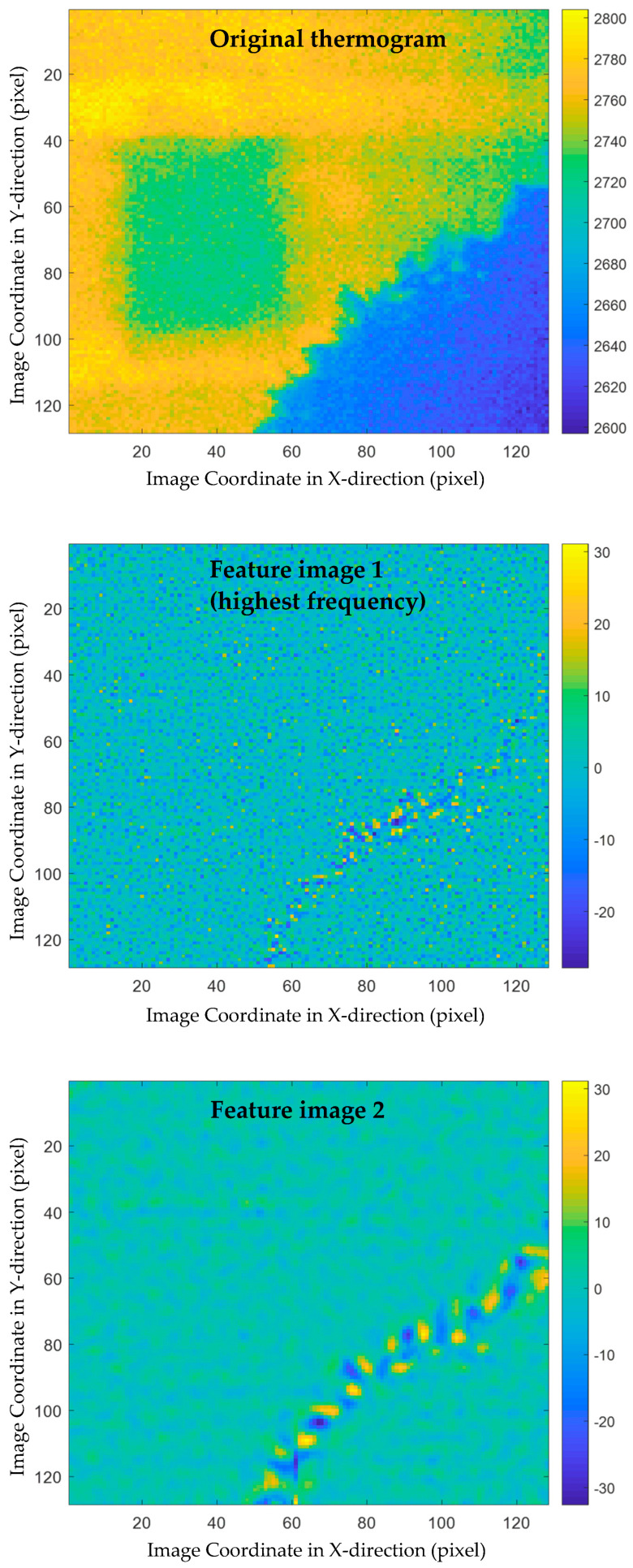

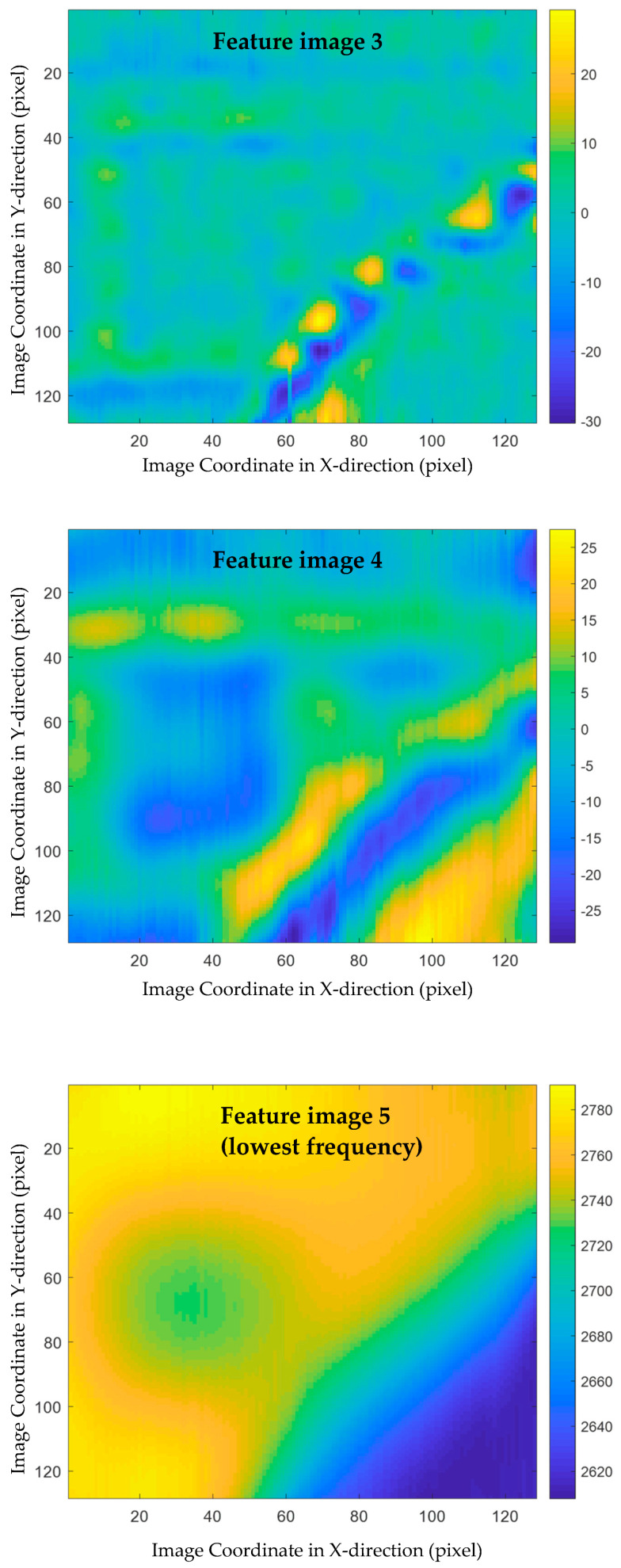
MEEMD results of a thermogram, i.e., Figure 4a: the original thermogram is plotted on the top followed by the feature images arranged from the highest spatial frequency to the lowest.

**Figure 6:**
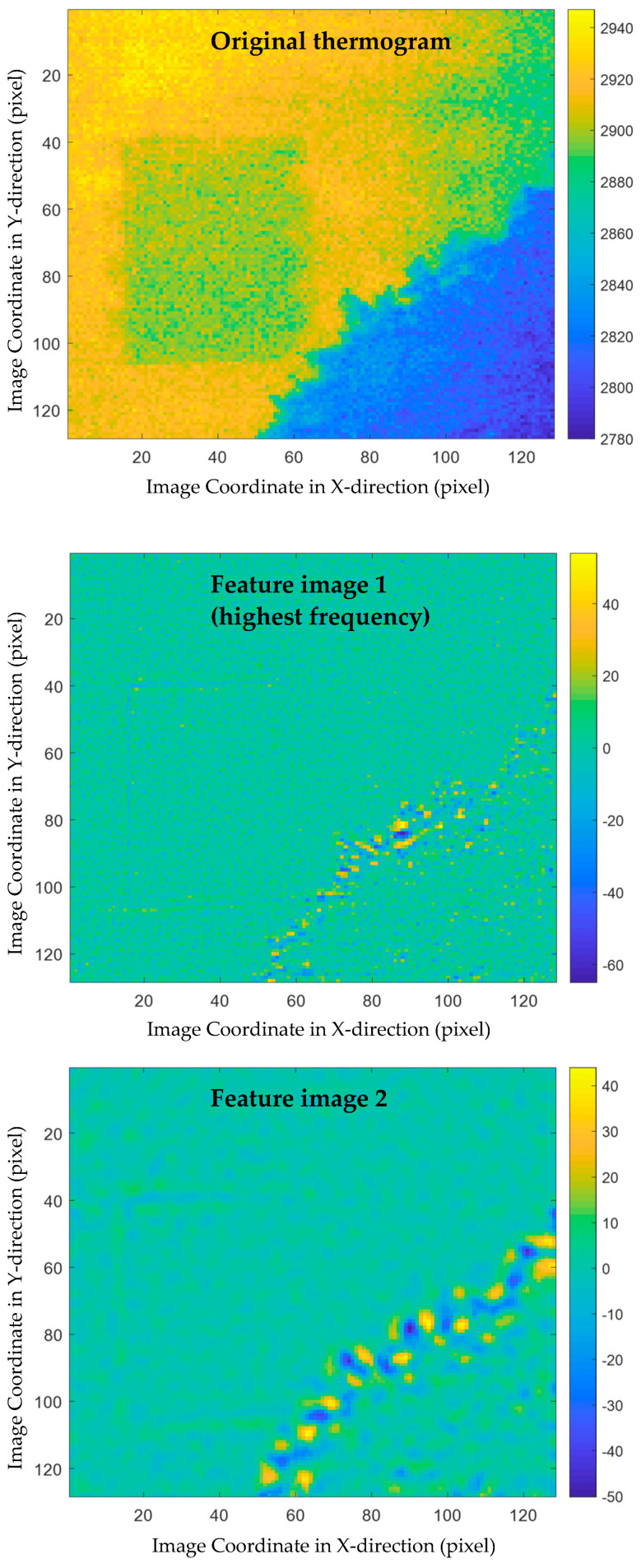

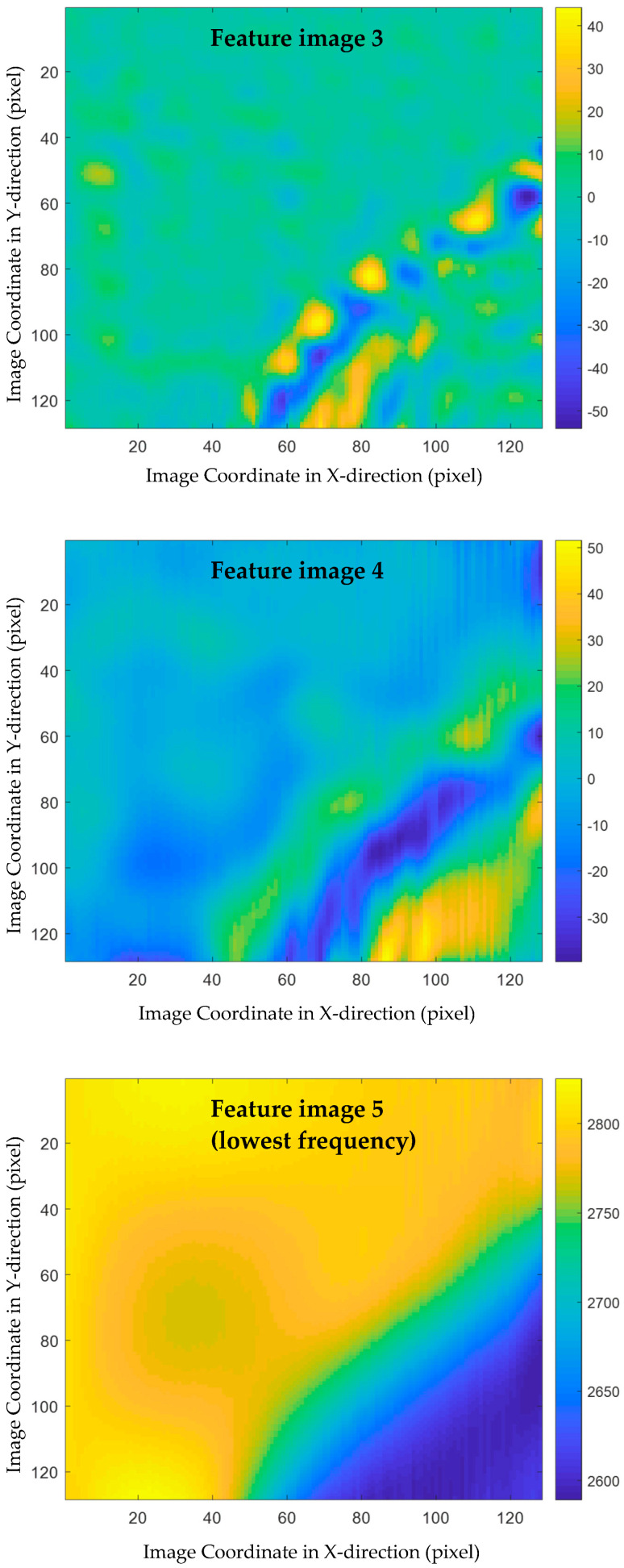
MEEMD results of another thermogram, i.e., Figure 4b: the original thermogram is plotted on the top followed by the feature images arranged from the highest spatial frequency to the lowest.

The decomposition results of two thermograms, i.e., the thermograms plotted in the first and second subplots of Figure 4, are shown in Figure 5 and Figure 6, respectively. In these two figures, the original thermograms are plotted on the top followed by the feature images arranged from the highest spatial frequency to the lowest. From both figures, it is observed that the feature image corresponding to the highest spatial frequency is the noisiest. The visual observation of this image cannot reveal much structural information. In contrast, the feature image with the lowest spatial frequency is the smoothest. At the middle spatial frequency band, some civil structures can be identified. A total of 190 feature images were obtained by applying MEEMD. It is laborious to investigate each of the feature images manually because of the large amount of them. In addition, it is difficult to explore the details of all feature images and distinguish between the interested structures and the surroundings. Therefore, further data compression and feature extraction is a desired step for post-processing the MEEMD results.

### 3.3. Multiscale Data Processing

Next, PCA was adopted to further disclose the structural features at different spatial scales. As introduction in Section 2.3, matrices $X_{k}$ (k=0,…, 5) were constructed, where $X_{0}$ contains the vectorized raw thermograms and $X_{1}$ to $X_{5}$ are composed of the vectorized MEEMD feature images at different spatial scales. Then, PCA was implemented to each of the matrices and the loading vectors were visualized.

**Figure 7:**
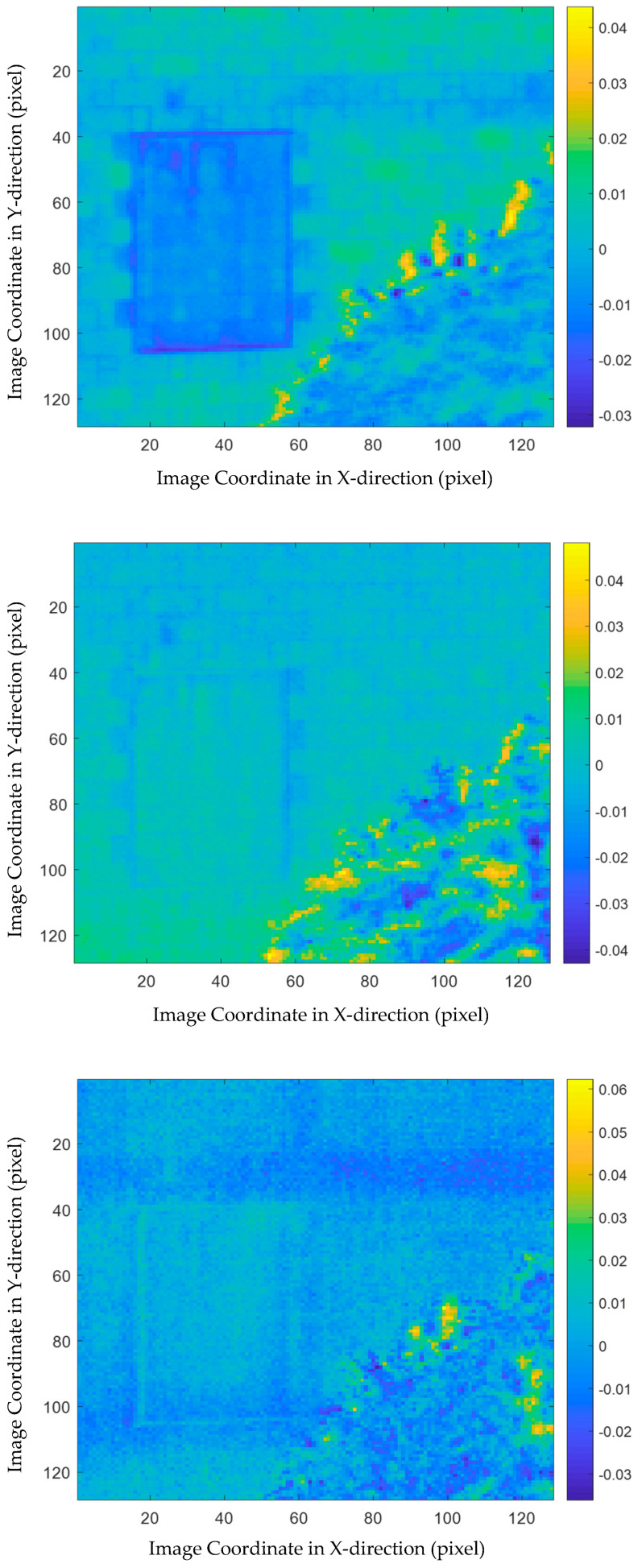
PCA loading images of $X_{0}$, i.e., PCT results.

Figure 7 shows the visualized PCA results of $X_{0}$ which are equivalent to the PCT results. In this figure, the first three loading images are plotted from the top to the bottom. From the first loading, i.e., the subplot on the top of Figure 7, the sealed door and the tree in the foreground can be identified, while the slab can also be observed vaguely. The second and third loading images do not provide much more information, although the third loading shows the slab more clearly. It is noted that the gray level difference or the contrast between the interested civil engineering structures and the backgrounds is not very significant, while the surface features, such as the ceramic tiles, are not very clear in this figure.

**Figure 8:**
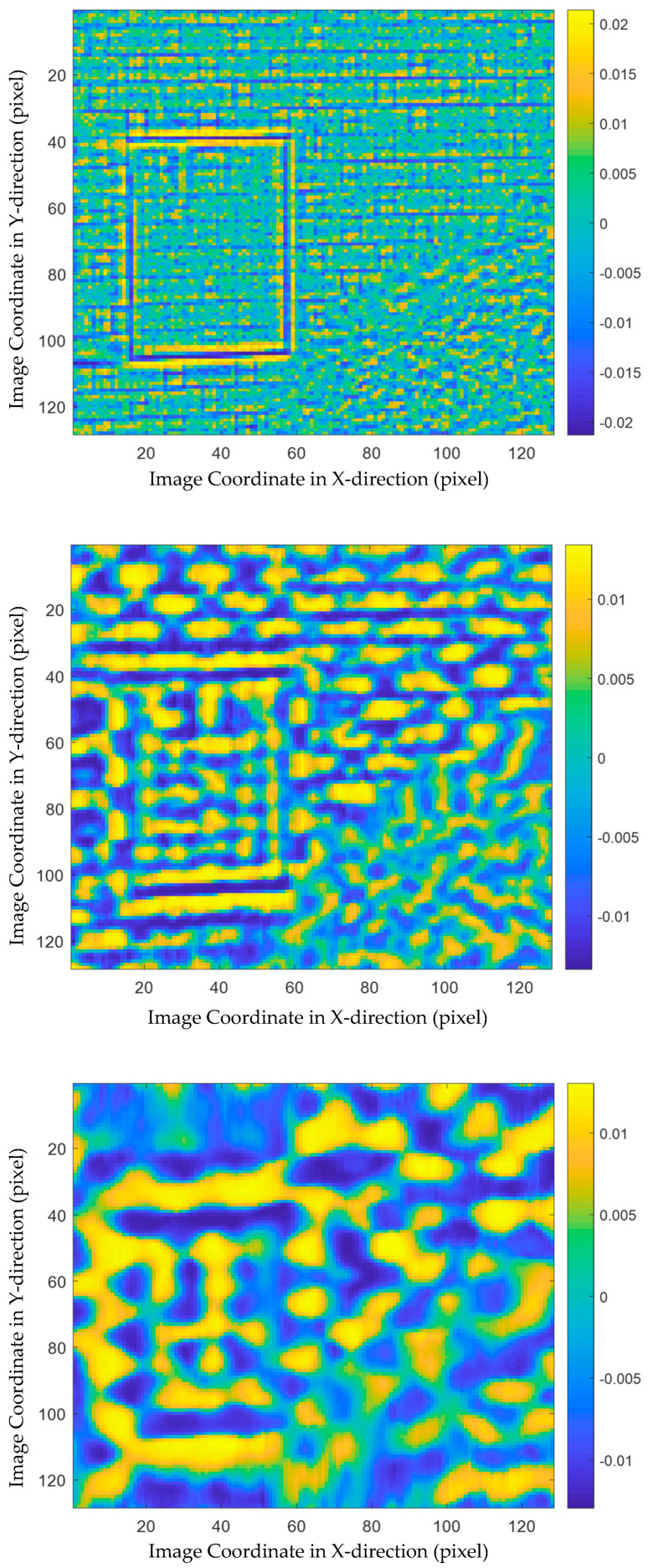

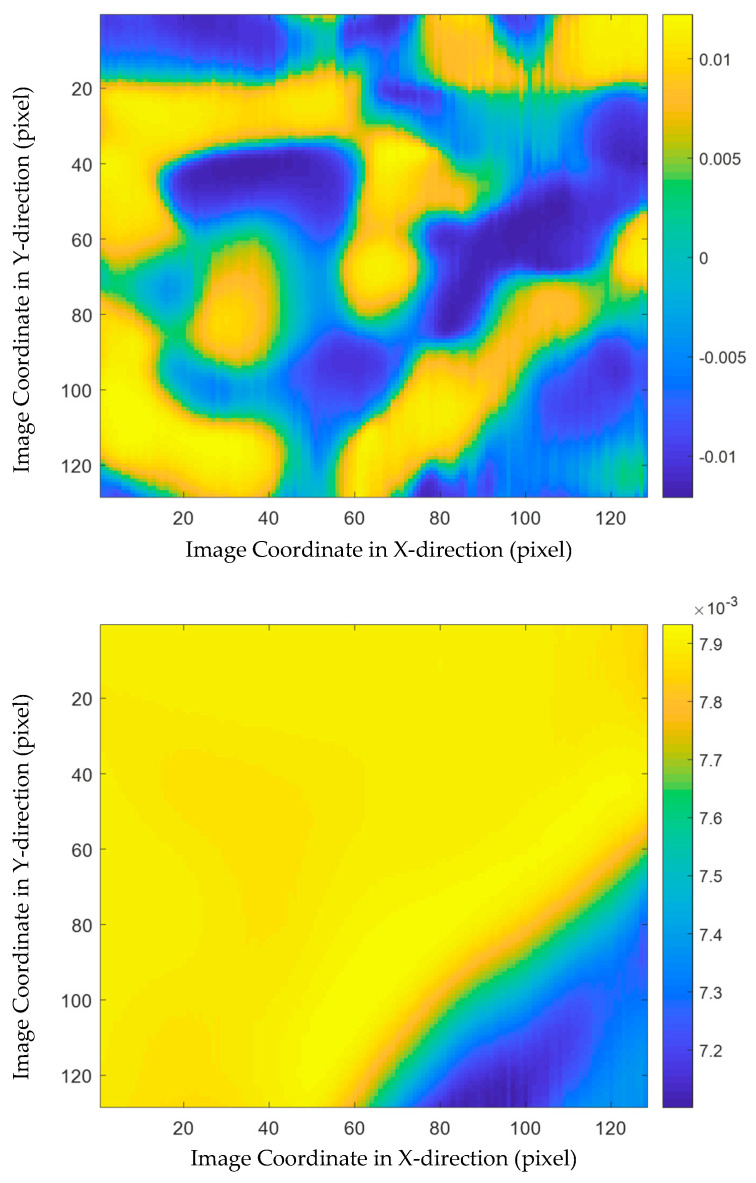
The first PCA loading images at different spatial scales.

The multiscale analysis results are a valuable supplement to the conventional PCT. Figure 8 shows the first loading images at different spatial scales. In other words, the five subplots in this figure visualize the first PCA loadings of $X_{1}$ to $X_{5}$, respectively. In the first subplot, i.e., the result achieved from $X_{1}$, many details of both surface and inner structure information are explored. Specifically, the frame of the sealed door is very clear because of the high gray level contrast around it. In addition, the tile joints are also highlighted in this image, which are not clearly identified in the loading images of $X_{0}$. The patterns in the loading image of $X_{2}$, which corresponding to the spatial scale of the second highest frequency, relate to the shapes and materials of the tiles. The third and fourth subplots do not provide much useful information, while the loading image of $X_{5}$ distinguishes the foreground, i.e., the region corresponding to the tree, at the lowest spatial frequency.

## 4. Conclusions

In this work, a thermographic data analysis method is proposed by integrating the strengths of multi-dimensional ensemble empirical mode decomposition (MEEMD) and PCA. MEEMD divides the information contained in each thermogram to several spatial scales corresponding to different frequencies, while PCA reduces the dimensionality of the MEEMD results and summarizes the results by feature extraction. The proposed method can be regarded as an extension of the conventional PCT method. Following the steps proposed in this paper, multiscale thermographic data analysis not only provides the PCT results, but also gives an opportunity to zoom in on different types of structural features and discovers more details of the civil engineering structures of the investigated object. The feasibility of the proposed method was illustrated with an experiment of building wall inspection based on solar loading thermography.

## Data Availability

Data are available upon reasonable request.
